# 

**Published:** 1878-11

**Authors:** 


### Table of Contents.

ORIGINAL COMMUNICATIONS.

Operation for Stricture, Followed by Uræmic Poisoning. *By H. L. Wilson, M.D., Atlanta, Ga*.......... 449
Cerebro-Spinal Meningitis. *By J. G. Westmoreland, M.D*.......... 451

REPORTS OF SOCIETIES.

Atlanta Academy of Medicine.......... 454
Proceedings of the Suffolk District Medical Society.......... 456
The Philadelphia Pathological Society.......... 461
Atlanta Medical College Clinic.......... 466

SELECTIONS.

The Local Treatment of Eczema.......... 471
Microscopical Observations in Yellow Fever.......... 480
Intra-ocular Circulation; Rhythmical Changes in the Venous Pulse of the Optic Disk.......... 483
Some Thoughts on the Therapeutics of Parturition.......... 487
The Theory of Reproduction.......... 490
Chronic Chloral Poisoning.......... 495
Cardiac Embolism following Delivery.......... 499

EDITORIAL.

The Yellow Fever Commission.......... 503
The International Decimal System of Weights and Measures.......... 504
American Pharmaceutical Association.......... 506

BIBLIOGRAPHICAL.

A Clinical History of the Medical and Surgical Diseases of Women.. 506
Illustrirte Vierteljahrsschrift der Ærztlichen Polytechnick.......... 507

EXCERPTA.

Chloral in Dysentery.......... 507
Acute Bright's Disease Cured by Jaborandi.......... 508
Venesection as an Anti-Hemorrhagic.......... 508
Questions of Maternal Influence.......... 509
The Thermal Death Point of Septic Organisms.......... 510
Icteric Urine.......... 511
Pessaries.......... 511
The Treatment of Diarrhœa by Oxide of Zinc.......... 512
The Action of the Tincture of Iodine upon the Uterine Neck.......... 512

